# Hyperspectral image analysis techniques for the detection and classification of the early onset of plant disease and stress

**DOI:** 10.1186/s13007-017-0233-z

**Published:** 2017-10-10

**Authors:** Amy Lowe, Nicola Harrison, Andrew P French

**Affiliations:** 1 School of Biosciences, University of Nottingham, Sutton Bonington, LE12 5RD UK; 2 School of Computer Science, University of Nottingham, Jubilee Campus, Nottingham, NG8 1BB UK; 3 NIAB EMR, New Road, East Malling, Kent, ME19 6BJ UK; 4 Agriculture and Horticulture Development Board, Stoneleigh Park, Kenilworth, Warwickshire CV8 2TL UK

**Keywords:** Hyperspectral imaging, Image analysis techniques, Vegetation Indices, Plant disease and stress, Early detection of stress, Hyperspectral image analysis

## Abstract

This review explores how imaging techniques are being developed with a focus on deployment for crop monitoring methods. Imaging applications are discussed in relation to both field and glasshouse-based plants, and techniques are sectioned into ‘healthy and diseased plant classification’ with an emphasis on classification accuracy, early detection of stress, and disease severity. A central focus of the review is the use of hyperspectral imaging and how this is being utilised to find additional information about plant health, and the ability to predict onset of disease. A summary of techniques used to detect biotic and abiotic stress in plants is presented, including the level of accuracy associated with each method.

## Background

The reliable detection and identification of plant disease and plant stress are a current challenge in agriculture [4, 5]. Standard existing methods of detection often rely on crop agronomists manually checking the crop for indicator signs that are already visible. Depending on the type of crop and the size of the crop area–which for many commercial crops is often very large–this method of monitoring plant health is both time consuming and demanding. Manual detection also relies on the disease or stress exhibiting clearly visible symptoms, which frequently manifest at middle to late stages of infection. Identification of the causal agent is through either manual detection or diagnostic tests [6]. Diseases usually start in a small region on the foliage (e.g. *Septoria tritici* blotch (STB) of wheat caused by the fungal pathogen, *Mycosphaerella graminicola*; Apple scab caused by *Venturia inaequalis*), which can be difficult to detect by visual inspection if the crop is large; however, the ability to identify the disease at this early stage would provide an opportunity for early intervention to control, prevent spread of infection, or change crop management practices before the whole crop is infected or damaged. Identifying crop areas affected by disease could also lead to targeted application of chemicals. Such precision approaches would result in the reduction of pesticide and herbicide usage, with subsequent beneficial impact for the environment, ecosystem services, grower finances and the end consumer. Hence, there is a keen interest in the agricultural and horticultural sector to replace this largely manual process with more automated, objective, and sensitive approaches. Mahlein has discussed the literature on plant disease detection by imaging sensors. This includes RGB, Multi spectral, Hyperspectral, thermal, Chlorophyll Fluorescence and 3D sensors. One conclusion is that RGB and hyperspectral imaging are preferable for identifying specific diseases [7].

To improve crop management and plant health, several avenues of research are focussing on the identification of the onset of adverse stresses, ideally before visible signs are present. Image analysis techniques show much potential here as they represent non-invasive and potentially autonomous approaches to detect biotic and abiotic stress in plants. This is illustrated in a recent review by Singh et al. [8] which examines machine learning for stress phenotyping, exploring literature on high through-put phenotyping for stress identification, classification, quantification and prediction using different sensors.

Image analysis as a research field represents a host of computational techniques which are able to extract information from digital images. From a practical point of view, this means automatic processing of carefully captured images to produce a dataset of desired measurements from the images. The images themselves can come from a variety of sources, from colour digital cameras or smartphones, to more specialist cameras designed to capture a variety of different information in the images. One such technological advance here is hyperspectral imaging, where cameras capture more than the usual three bands of coloured light found in traditional digital imaging. This review will specifically focus on the subsequent analysis approach known as hyperspectral image analysis. This approach has recently become financially accessible to a wide variety of users, due to falling technology costs. Analysis approaches are being developed which are enabling the Hyperspectral imaging technologies to be utilised for wider ranging applications. Hyperspectral imaging uses high-fidelity colour reflectance information over a large range of the light spectrum (beyond that of human vision), and thus has potential for identifying subtle changes in plant growth and development.

In this review, we provide an overview of hyperspectral imaging, and how it can be utilised in laboratory and field applications for the categorisation and recognition of early stages of plant foliar disease and stress. Starting with the background theory and an overview of Hyperspectral imaging technology, we then consider some areas of application of the approach to plant and crop sciences. Finally, we discuss some practical concerns with these approaches; an important aspect, as such cameras are not yet typically provided as a turnkey solution for crop monitoring, so care must be taken to collect satisfactory data and provide meaningful analysis and interpretation before deployment of these technologies can be implemented in a commercial setting.

### Colour digital imaging

In order to understand the hyperspectral technology itself, it will be helpful to first consider what a standard, non-hyperspectral colour digital image comprises. Wavelengths of light correspond to colour, with blue light having a central wavelength of approximately 475 nm, green light 520 nm, and red light 650 nm. A colour image represents a composition of three broad wavelength bands, red, green and blue. Our eyes contain three types of cones, sensitive to blue, green and red parts of the spectrum, the cones each have a colour range and they are stimulated either strongly or weakly depending on the light wavelengths emitted. Combining the information from the three different kinds of cones we recreate a colour image in our brain. A digital image tries to emulate the sensitivity of the cones, and a pixel stores the integrated intensity of either the blue, green, or red part of the light spectrum, dependant on the filter type placed in front of the pixel.

**Fig. 1:**
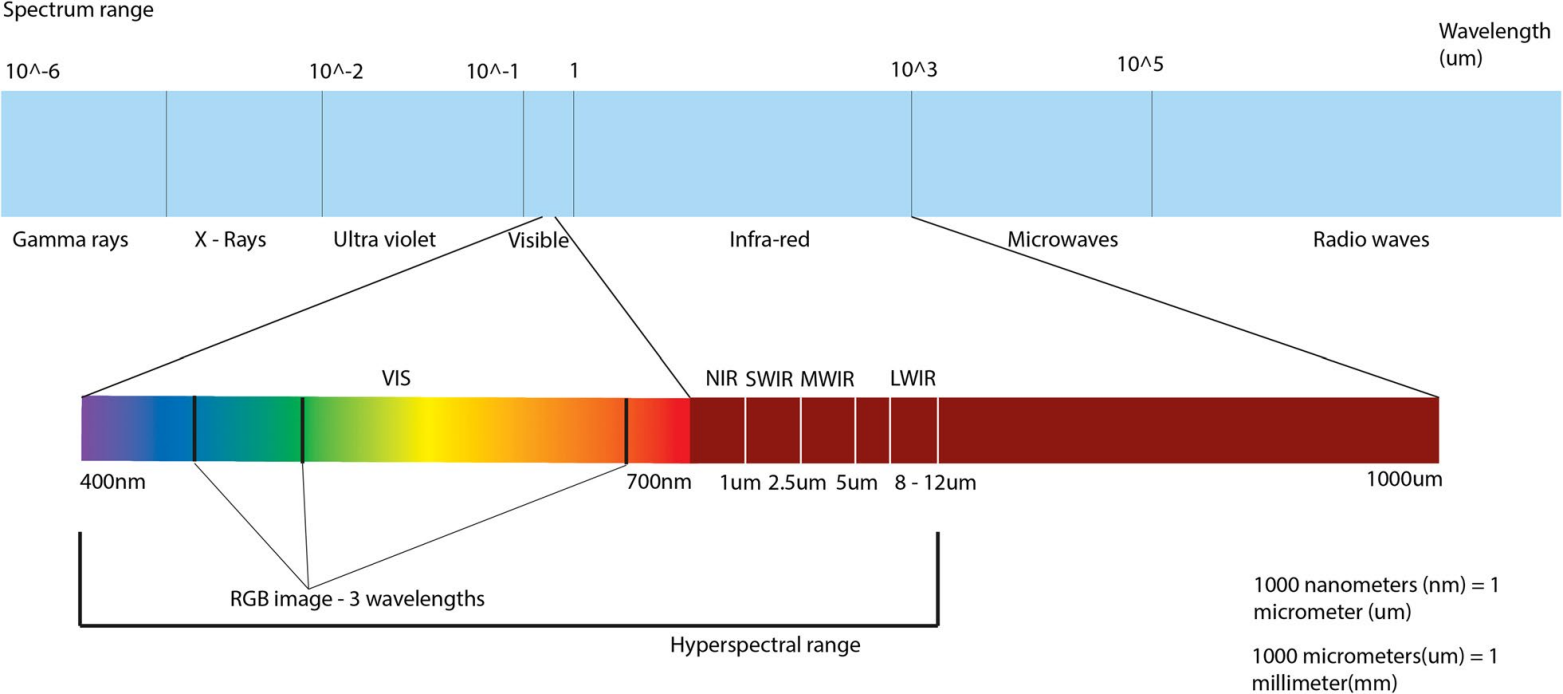
Electromagnetic spectrum with the lower bar displaying visible and infra-red light

The range of light captured in a hyperspectral system can also vary. The colour visible to the human eye is a small range on the electromagnetic spectrum, ranging from 400 to 700 nm (Fig. 1). The section of the spectrum that is typically used for hyperspectral imaging of plants ranges from ultraviolet (UV) (starting at ~ 250 nm) up to short-wave infrared (SWIR, ~ 2500 nm). Cameras usually capture a certain sub-range, such as the visible and near infrared range (VIS–NIR, 400–1300 nm) or the SWIR (1300–2500 nm) or UV (250–400 nm) with the ranges being combined in some sensors to increase the coverage of the spectrum.

A colour image, then, is an example of a 3-band *multispectral* image, where each band records one of the three colours, red, green and blue. It is common to have more bands in a true multispectral image, perhaps sampling light in the infrared region of the spectrum too—that is, light with a wavelength over 700 nm. Hyperspectral images on the other hand typically contain hundreds of contiguous narrow wavelength bands over a spectral range. The approach produces a dense, information-rich colour dataset, with enough spatial resolution to have many hundreds of data points (pixels) per leaf.

For plants and vegetation the most useful wavelength ranges to analyse are the visible range combined with near infrared range. This wavelength range can capture changes in the leaf pigmentation (400–700 nm) and mesophyll cell structure (700–1300 nm) however to see changes in the water content of a plant, extended ranges are needed (1300–2500 nm) [9]. Severe dehydration, for example, can affect the leaf mesophyll structure which relates to changes in the near infrared reflectance; however, minor drought stress does not usually have enough of an effect to be detected [10].

### Hyperspectral imaging technology

There are various hardware approaches behind hyperspectral imaging spectrometers, which means there are different ways that the image is captured. Examples of operation include push broom, filter wheel, liquid crystal tunable filters amongst others [11]. In one example using push broom, the incoming light passes through a convex grating (or a prism) which separates the light into narrow wavelengths. This separation is then recorded on a light sensitive chip (similar to a standard digital camera). A push broom device, has three components; the camera, a spectrometer and a lens. This system simultaneously captures a single spatial line of the image, and the whole colour spectrum range. Then the camera or object is moved and the next line is captured (the broom is ‘pushed’ forwards, hence the name), effectively making the camera a line scanner, with the final image being built up after the full scan is complete. An alternative to push broom is a snapshot approach, where the entire image is captured at once. To date, push broom technology has seen the most use, but recent advances in snapshot technology are increasing the uptake and possibilities related to phenotyping and analysis.

In the rest of this review, we consider applications of the hyperspectral imaging technology and analysis, and have categorised the review into the following four sections: (1) existing vegetation and disease indices; (2) applications for the detection and classification of healthy and diseased plants with disease classification; (3) quantifying severity of disease; and (4) early stage detection of stress symptoms.

Within these sections, we will consider both laboratory-based imaging approaches, and field-based remote sensing. As well as the obvious biological differences, it is worth considering the impact of these environments on the hyperspectral image data itself. Laboratory-based imaging occurs in a controlled environment which includes artificial light. Outdoor remote sensing data is often dependant on ambient illumination, although there are examples of systems using controlled lighting for outdoor hyperspectral imaging [12]. Using natural illumination, namely the sun, means recognising that there are atmospheric effects such as the absorption and scattering of light. Other environmental factors that can contribute to a change in the spectral signatures are the interaction between cloud shadows and the object’s surface, time of day, specular reflections and the presence of other objects that can reflect secondary illumination onto the area of interest. As many of these effects are time dependant, successful use of a calibration reference means updating the referencing whenever ambient illumination changes—this could be minute to minute in a natural illumination scenario. With controlled lighting there are still problems; light intensity challenges exist: the inverse square law states that illumination drops off inversely according to distance from the light source [13]. This means that uneven illumination can occur and the type of light source chosen needs careful consideration; it should not have high intensity peaks throughout the spectrum or across the image plane.

Another potential difference between laboratory and field imaging is resolution. For aerial remote sensing data, the spatial resolution is typically in the range of meters per pixel, which means the pixels will usually contain signatures from more than one material [14, 15]. A first step in analysing this data is to consider this multi-material problem, whereby pixels must be considered to contain mixed materials (called ‘mixed pixels’) [16, 17], and a spectral unmixing process must be applied. In other words, a single pixel may contain plant and soil, and algorithms must be used to determine the appropriate mix. In the laboratory, images can typically be taken within centimetres of the plant, and there may be many pixels representing even a single leaf or region of disease. In these cases, unmixing is generally not necessary.

Further consideration of these location-based challenges will be fully explored later in this review, but before we continue let us consider why we wish to capture such hyperspectral information in the first place.

## Applications for the detection and classification of healthy and diseased plants

In this section, we will discuss a variety of techniques used specifically for the detection of biotic stress in plants. Classification techniques, that is, techniques that separate the data into healthy and diseased categories for example, can be divided into two types: those that focus on a number of key wavelengths in the spectrum and those that use the entire spectrum response. Furthermore, disease classification is discussed with regards to the identification of multiple diseases and detection of a specific disease.

### Existing vegetation and disease indices

Before hyperspectral imaging devices were readily available, researchers wishing to quantify effects based on colour information have used multispectral imaging, or hyperspectral, point-source devices (such as spectroradiometers which do not produce a spatial image) to acquire colour data. Hyperspectral devices do not in general provide a point-and-click measurement. Instead, much onus is on the user to develop the capture process. Once acquired, the resulting large numerical datasets must be analysed in order to provide useful information. A sensible and simple way into such large datasets is to consider only a small number of positions in the wavelength range, looking at changes across conditions at predetermined key points in the spectrum. Using this approach, we can also counter the effects of relative light changes by considering ratios of data values. This involves the combination of two or more wavelengths, commonly known as ‘indices’.

To interpret the data, a number of such indices have been developed, through either pre-considered biological reasoning (e.g. knowing that a particular wavelength relates to properties in a particular cell structure), or due to limitations in the particular wavelengths available from the capture equipment (e.g. indices which are derived from satellite multispectral remote sensing data may only have had a limited number of wavelengths available). When applied to plant material, these indices are known as ‘vegetation indices’. Many different vegetation indices exist and each uses a different set of wavelength measurements for describing physiological attributes of vegetation, looking at either general properties of the plant, or at specific parameters of its growth.

One of the most popular and widespread metrics is the normalised difference vegetation index (NDVI), which is used for measuring the general health status of crops [18, 19]. It is calculated via a simple ratio of near-IR and visible light (see Table 1). NDVI has been used for many different purposes, for example, to detect stress caused by the Sunn pest/cereal pest, *Eurygaster integriceps Put.* (*Hemiptera: Scutelleridae*), in wheat [20]. Most of the indices are very specific and only work well with the datasets that they were designed for [21]. There are disease-centric studies focused on creating disease indices for detecting and quantifying specific diseases [22], for example, one study used leaf rust disease severity index (LRDSI) with a 87–91% accuracy in detecting the leaf rust (*Puccinia triticina*) in Wheat [23], however, to our knowledge, it has not been widely tested.

**Table 1:** A selection of vegetation indices

VI	Formula	References	Information
Normalised difference vegetation index (NDVI)	(RNIR − RRED)/(RNIR + RRED) RRED ~ 680, RNIR ~ 800	[50]	Range: − 1 to 1 Common range: 0.2–0.8 Broadband
Red edge NDVI	(R750 − R705)/(R750 + R705)	[50]	Range: − 1 to 1 Typical healthy range: 0.2 to 0.9 Narrowband (hyperspectral data)
Simple ratio index (SRI)	RNIR/RRED RRED ~ 680, RNIR ~ 800	[20]	Range: 0 to > 30 Typical healthy range: ~ 2–8 Broadband
Photochemical reflectance index (PRI)	(R531 − R570)/(R531 + R570)	[50] [51]	Range: − 1 to 1 Typical healthy range: − 0.2 to 0.2 Vegetation health prior to senescence
Plant senescence reflectance index (PSRI)	(Red–Green)/NIR	[50]	Range: − 1 to 1 Typical healthy range: − 0.1 to 0.2 >PSRI ~ canopy stress, onset of senescence, fruit ripening
Normalised phaeophytinization index (NPQI)	(R415 − R435)/(R415 + R435)	[52]	Chlorophyll degradation 0.56–1.41 Unacidified and acidified solutions [53]
Structure Independent Pigment Index (SIPI)	(R800 − R445)/(R800 + R680)	[50] [51] [54]	Range: 0–2 Typical healthy range: 0.8–1.8 Good with canopy variety
Leaf rust disease severity index (LRDSI)	6.9 × (R605/R455) − 1.2	[23]	Accuracy of 89% in study may vary with other data.

**Fig. 2:**
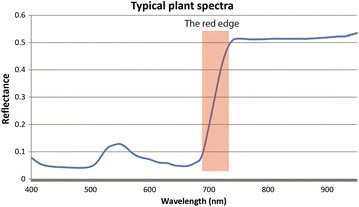
A typical healthy vegetation spectra (400–1000 nm) with the red edge section highlighted in red (690–740 nm)

Another commonly-used approach is to detect changes in the sudden increase in reflectance at the red/near-infrared border. This ‘red edge’ position is a narrow section in the electromagnetic spectrum (690–740 nm) where the visible spectrum ends and the near infrared starts (Fig. 2). This section has a large change in spectral response (derivative),for green plant material, since chlorophyll strongly absorbs wavelengths up to around 700 nm, and hence the material has low reflectance in this range, but it is strongly reflecting the infrared (from about 720 nm). Cho [24] describes a number of different algorithms that extract or detect the red edge. A disease index based on the red edge position has been used to detect powdery mildew in wheat (*Blumeria graminis* f. sp*. Tritici*), however it was not as accurate as Partial Least Squares Regression (PLSR), a technique that uses a statistical approach [25]. We will consider some of these statistical approaches further in this review.

### Classification using a subset of selected wavelengths

In this section we consider classification approaches that rely on sub sampling at particular wavelengths from the full spectrum. One difference with true multispectral data is that specific wavelengths can be manually or automatically chosen from anywhere in the captured range, where as multispectral data is limited by the technology.

Analysis from “Background” section typically used indices to calculate representative values using discrete wavelengths at various positions in the spectrum. One such study involving a wheat field experiment used normalised difference vegetation index (NDVI) response to eliminate everything except the leaves from the dataset, followed by a statistical approach called an ANCOVA (which measures statistical covariance) to identify selected wavelength bands, and then quadratic discriminant analysis (QDA) to classify the spectra between healthy and diseased leaves (yellow rust) [26]. This is representative of a typical workflow in hyperspectral analysis: isolate (or segment) the parts of the image of interest, then use a mathematical technique to identify regions of the spectra likely to have predictive power, and finally use those spatial and spectral regions to learn a classification approach. Using QDA, the overall accuracy reached 92% with 4 wavebands [26].

An example of multi layer perceptrons (MLP) is described in Moshou [27], who aimed to detect yellow rust in field-grown wheat using a spectrograph with the range 460–900 nm and a 20 nm spectral resolution. The spectrograph captured the images in the field using a handheld system. Then four significant wavelengths were selected. The first two wavelengths were selected using ‘variable selection’ which involved comparing the wavelengths using stepwise discriminant analysis and using the F-test. The second pair of wavelengths uses the NDVI wavelengths. The neural network used by Moshou is a simple architecture with four inputs, one hidden layer consisting of ten neurons and two outputs (healthy and diseased). The architecture is determined by the number of inputs, a selected amount of hidden neurons and the amount of outputs required. Trial and error can be used to determine a suitable architecture. Moshou tried different quantities of neurons and selected the most efficient. The classification accuracy reached using this approach was 98.9% for the healthy plants and 99.4% for the diseased plants.

The MLP approach uses a simple architecture consisting of an input, hidden layer(s) and the output. In machine learning a new, more sophisticated approach called deep learning is becoming popular. Deep learning refers to artificial neural networks with a structure that contains a lot of layers, and during each layer neurons are able to implicitly represent features from the data and by doing this, more complex information can be obtained in later layers, and image features are automatically determined by the network. One specific example of a deep learning approach is convolutional neural networks (CNN). Whilst artificial neural networks (ANN) use neuron activation networks as their analogous model, CNNs are based on retinal fields in the vision system. Whatever the approach, deep learning takes longer to train and the architecture is more complex, however, with the added complexity, very impressive classification and recognition rates are achievable.

Deep learning has been applied to the problem of plant disease detection. Mohanty [28] used CNN’s to detect 26 diseases over 14 crop species. A dataset consisting of 54,306 colour images were used, 80% for training and 20% for testing on AlexNet and GoogLeNet (two popular versions of pretrained CNN’s). The accuracy was 97.82% for AlexNet and 98.36% for GoogLeNet using colour images with training from scratch (for transfer learning the values are higher, 99.27 and 99.34% respectively). They selected individual leaves with a homogenous background. If the network is tested on images under different conditions from the trained images the accuracy is 31.4% [28]. Sladojevic also used CNN’s to detect 13 diseases across various crop plants, including Apple (powdery mildew, rust), pear (leaf spot), grapevine (wilt, mites, powdery mildew, downey mildew) using 30,000 images with an accuracy of 96.3% using CaffeNet [29].

There are currently very few complete studies applying deep learning to hyperspectral data, though this is an active research area. There are several challenges that need to be addressed in order to use hyperspectral data for deep learning. The size of the hyperspectral data including the amount of wavelengths would require a lot of processing time and power it would ideally require a graphics processing unit. The amount of hyperspectral wavelengths would most likely include noise from specific wavelengths. Also there needs to be a sufficient amount of data for the training/testing process along with labelled data. There is also the possibility that the error will be higher than alternative approaches.

Other non-deep learning approaches include Yuan [30], using Fishers Linear Discriminant Analysis with remote sensing data to detect yellow rust and powdery mildew for a wheat crop with an overall accuracy of 93% with selected wavelength ranges (531, 570–654, 685–717 nm) that are significant for detecting differences between powdery mildew and yellow rust diseases in these spectral reflectance ranges, resulting from an independent *t* test.

Sometimes data analysis approaches are combined with simple image processing steps in order to add feature discrimination. A family of image processing techniques called morphological operators can be used to clean up binary (black and white) images. One such technique is called erosion, whereby the foreground of an object is shrunk by turning boundary pixels into background pixels. The opposite technique is called ‘dilation’ and has the effect of enlarging the foreground object’s boundary. They can be used together to fill in holes, or remove speckle noise (depending on the order used) in binary labelled data. One approach using this method is a study on cucumber leaf data, in this example, this technique has been used to analyse a different type of mildew; downy mildew (*Pseudoperonospora cubensis*). first principle component analysis (PCA) is applied to reduce the size of the data and a binary image is produced, and then erosion and dilation are used in a second step to enhance the disease features. The accuracy is 90% however only 20 samples were used (10 healthy and 10 infected) [31]. This method is unlikely to work as well on other hyperspectral images to detect diseases unless the leaf data is similar and even then the results are uncertain.

Hyperspectral imaging can also be combined with microscopy to capture images at a higher resolution. Barley with different genotypes has been studied at the microscopic level to see if spectral differences could be identified between the genotypes. Barley leaves were also analysed from both healthy and diseased plants, which had been inoculated with Powdery Mildew (*B. graminis*). Results showed there was a difference over time between the healthy and inoculated leaves, except for those varieties containing the mildew locus o (mlo) gene, which provides plant resistance to *B. graminis.* In this study, the spectral range was reduced to 420–830 nm due to the noise, then normalised and smoothed with Savitzky-Golay filter, and then SiVM is used to find the extreme spectra followed by Dirichlet aggregation regression for the leaf trace [32].

### Classification using full spectrum data

Classification approaches aim to divide the data into a number of distinct classes. They originate from a family of statistical or machine learning techniques. One such approach is quadratic discriminant analysis (QDA), which classifies by using a covariance matrix, which compares classes. The QDA method was used in a study with Avocado plants, to examine the fungal disease Laurel wilt (*Raffaelea lauricola*), using plants located both in the field and glasshouse. The QDA classification accuracy was 94% [33]. It is possible of course to use alternative methods at each stage of the analysis pipeline. For example, rather than use QDA, a decision tree approach (a machine learning technique) has been used and reached 95% accuracy [33]. Choosing the correct approach for the data, as well as ensuring sufficient dataset size and quality, is key. Such machine learning approaches represent an increasingly-common set of classification and prediction algorithms. Machine learning approaches train algorithms using a training dataset, with the aim of analysing and predicting results from new, unseen data. Multilayer perceptron’s (MLP) are an example of such a technique. MLP’s are simple networks (called artificial neural networks) that maps input data to an output. This process is based on biological understanding of neuron activation networks where messages are fired between neurons. The input node connects to the output and it is updated using an activation function and weights that can be optimised to produce the correct output (using training data). This algorithm requires prior knowledge (training data) therefore if the ‘disease spectra’ is unknown then this technique will be unsuitable.

A third classification approach is to look at the spectral signatures by using derivatives; this is when the underlying pattern or change in data is analysed. Second order (and above) derivatives are usually insensitive to changes in the illumination [15]; however they are sensitive to noise which hyperspectral data typically suffers from, therefore ‘smoothing’ needs to be applied before using derivatives. Smoothing is a process that reduces the difference between individual pixel intensities and neighbouring pixels using forms of averaging to create a smoother signal. Two smoothing examples are Savitsky-Golay and Gaussian filtering. Savitszky-Golay proposed a method for smoothing noisy data by fitting local polynomials to a sub set of the input data then evaluating the polynomial at a single point to smooth the signal [34]. Gaussian filtering reduces noise by averaging the spectral data with a focus on the central information using a Gaussian-weighted kernel.

Huang [35] tries to detect Sclerotinia rot disease in Celery crops by using partial least squares regression (PLSR) with derivatives of first and second order. Partial least squares regression selects a small set of components. This technique is useful when the predictors are collinear/highly correlated, and it will reduce the risk of overfitting the data. The classification accuracy for Partial least squares regression with the raw spectra is 88.92%, PLSR with Savitzky-Golay first derivative is 88.18% and PLRS with second order derivative is 86.38%. The accuracies are similar, with the second order derivative performing slightly worse. Yuan [36] uses PLSR on Fisher’s linear discriminant analysis (FLDA) to detect pest and disease in wheat. It produced a 60% accuracy for aphid damage and a 92% accuracy for Yellow rust disease. In another study, Zhang [37] used FLDA to detect powdery mildew in wheat (using a heavily damaged leaf) with over 90% accuracy.

### Disease identification

As well as detecting the *presence* of disease, another avenue of research is to distinguish between *different* diseases to identify specific pathogens. One such approach is spectral information divergence classification. This method compares the divergence between the observed spectra and a reference spectra (a library of spectra, or average spectra of interest from the data), where the smaller the divergence value then the more similar the spectra are, and if they are larger than a set threshold then they are not classified as the reference spectra [3]. Spectral information divergence was used to detect canker legions on citrus fruit (grapefruits) where the spectral range of the data was 450–930 nm with 92 bands and 5.2 nm spectral resolution. Before analysing the data, a pre-processing step is applied by combining neighbouring pixels to reduce the size by half. Cankerous grapefruits were compared with normal grapefruit and also with grapefruit showing other disease or damage symptoms including: greasy spot, insect damage, melanose, scab and wind scar; this method resulted in 95.2% classification accuracy [38].

## Quantifying severity of disease

Along with detecting and classifying disease, we may wish to record the effective amount of disease, or its severity. This approach does run into some particular challenges. The amount of leaf damage and coverage from the disease can affect the accuracy of the leaves being classified as healthy or diseased. Extreme disease damage can affect the appearance of leaves so detrimentally that they may not be counted as plant material at all. Still, there are a number of methods for estimating severity, and we present a selection of approaches below.

Spectral angle mapper (SAM) approaches match the pixel spectra to reference spectra to classify the pixels by calculating the angle between the spectra which are treated as *n*-dimensional vectors in space [2]. This technique has been widely used with moderate success to classify hyperspectral data, including plant diseases. Yuhas studied the severity of *Fusarium* head blight disease for wheat before harvesting. The hyperspectral data was in the range 400–1000 nm with a spectral resolution of 2.5 nm. SAM was used to detect the *amount* of disease with a classification accuracy of 87%. Two experiments with wheat plants were carried out, one in a glasshouse and one in field. The plants were imaged over their developmental stages from inoculated to established infection. Yuhas determined that just after infection, the healthy and infected plants were not distinguishable because the infection had not yet established. However, when the hyperspectral data were examined during the ripening stage, the wheat pigment composition changes, and the healthy plants then appear as diseased plants [39].

Mahlein [40] uses the same technique to analyse sugar beet diseases specifically *Cerospora* leaf spot, powdery mildew and leaf rust. The range is 400–1000 nm with 2.8 nm spectral resolution and 0.19 mm spatial resolution. The plants were analysed over a time period (> 20 days) to monitor the different stages of each disease, and the leaves were classified as healthy or diseased. *Cerospora* leaf spot classification accuracy varied depending on the severity of the disease (89.01–98.90%), powdery mildew accuracy varied between 90.18 and 97.23%, and sugar beet rust reached 61.70%, with no classification before day 20 using SAM.

Rumpf et al. [41] used the same dataset as Mahlein but with different analysis approaches; decision trees (DT), artificial neural networks (ANN) and support vector machine (SVM). All approaches require prior knowledge, however once trained have proven to be efficient. For example, with *Cerospora* leaf spot the accuracy for SVM is 97% (compared to DT 95% and ANN 96%); for Sugar beet rust the accuracy is 93% (DT 92%, ANN 95%); and for Powdery mildew the accuracy is 93% (DT 86%, ANN 91%). Measuring the severity with leaf area coverage after the disease has covered 1–2% of the leaf the accuracy is 62–68% and for more than 10% leaf coverage the accuracy is almost 100%. This demonstrates that it is possible to use a variety of analysis methods on the same set of hyperspectral data to elucidate different insights and achieve different levels of accuracy—choice of technique is important. A list of common techniques used to identify specific diseases and the accuracy associated with each is presented in Table 2.

**Table 2:** Summary of techniques successfully used to detect drought and diseases in plants

Technique	Plant (stress)	References	Accuracy
Quadratic discriminant analysis (QDA)	Wheat (yellow rust) Avacado (laurel wilt)	[26] [33]	92% 94%
Decision tree (DT)	Avacado (laurel wilt) Sugarbeet (cerospora leaf spot) Sugarbeet (powdery mildew) Sugarbeet (leaf rust)	[33] [41]	95% 95% 86% 92%
Multilayer perceptron (MLP)	Wheat (yellow rust)	[27]	98.9/99.4% H/D
Partial least square regression (PLSR) Raw Savitsky-Golay 1st derivative Savitsky-Golay 2nd derviative	Celery (sclerotinia rot)	[35]	88.92% 88.18% 86.38%
Partial least square regression (PLSR) Fishers linear determinant analysis	Wheat (yellow rust) Wheat (aphid) Wheat (powdery mildew) Wheat (powdery mildew)	[36] [37]	92% 60% 90%
Fishers linear determinant analysis (FLDA)	Wheat (yellow rust) Wheat (powdery mildew)	[30]	93%
Erosion and dilation	Cucumber (downey mildew)	[31]	90%
Spectral angle mapper (SAM)	Sugarbeet (cerospora leaf spot) Sugarbeet (powdery mildew) Sugarbeet (leaf rust) Wheat (head blight)	[40] [39]	89.01–98.90% 90.18–97.23% 61.7% 87%
Artificial neural network (ANN)	Sugarbeet (cerospora leaf spot) Sugarbeet (powdery mildew) Sugarbeet (leaf rust)	[41]	96% 91% 95%
Support vector machine (SVM)	Sugarbeet (cerospora leaf spot) Sugarbeet (powdery mildew) Sugarbeet (leaf rust) Barley (drought)	[41] [45]	97% 93% 93% 10 days before visible signs
Spectral information divergence (SID)	Grapefruit (cankerous, normal, greasy spot. Insect damage, melanose, scab, wind scar)	[38]	95.2%
Simplex volume maximisation SiVM with DAR	Barley (drought) Barley (drought)	[44] [47]	4 days before Vegetation Indices 1.5wk Before visible signs
LSSVM	Wheat (drought)	[46]	86.6%(H)/76.3%(S)

*H* healthy, *S* stressed, *D* diseased

## Detection of early stage stress symptoms

The ultimate goal of such detection systems is to identify the disease with a minimum of physical changes to the plant. Identifying diseases or abiotic problems as early as possible has obvious benefits. By using hyperspectral technology in combination with appropriate analysis methods, we can realistically hope to identify stress symptoms before a human observer.

Drought can be a significant problem for many crops [42], particularly as some plant species or varieties do not visibly indicate this stress for a period of time, and by this time, the potential yield or quality of the crop may have decreased because normal plant developmental processes have been affected through the stress response. The definition of ‘drought’ can also vary from a little water deprivation to complete deprivation. Studies discussed in this section have detected the onset of drought before Vegetation Indices’ detected the drought and also days before visible signs appeared.

One technique in particular which has become popular for early detection of drought stress is simplex volume maximisation (SiVM), which is a data clustering technique [43]. This technique selects spectral signatures that are samples of healthy and stressed plants, and then clusters the data using these classes. When the signatures become similar to a pre-learned sample signature then it is classified as such.

Romer [44] studied drought stress in a barley experiment contained in a rainout shelter and a corn experiment grown in field. The technique used to detect the stress was simplex volume maximisation, which is an unsupervised technique. The spectrum range was 400–900 nm, with 4 nm spectral resolution. During pre-processing some wavelengths are removed due to noise (< 470 and > 750 nm). This is a common occurrence with hyperspectral data due to insufficient light at the end of the spectrum range, and is especially common with lab-based light sources which may not generate much light in these regions of the spectrum. To reduce the size of the data and to remove the background, a k-means clustering method was used to separate the data into a selected number of groups using mean colour. SiVM is then compared to four well known vegetation indices’—NDVI, photochemical reflectance index (PRI), red edge inflection point (REIP) and carotenoid reflectance index (CRI). For the Barley data, reduced partial water stress was detected four days earlier with SiVM (day 9) than Vegetation Indices’ (day 13). For the plants with no water/complete drought conditions the Vegetation Indices’ detected the stress on day 8, one day faster than SiVM, but they failed to detect the stress for days 9 and 10; however SiVM did reliably detect the stressed plants from day 9.

Behmann also analysed drought stress in barley using support vector machine (SVM). This algorithm is supervised and requires labelled training data, which in this case is labelled as drought or healthy. The data is pre-processed with k-means to reduce the size of the dataset before analysis with SVM. The spectral range was 430–890 nm with a spectral resolution of 4 nm. Using this approach, Behmann detected drought stress on day 6, with NDVI detecting a difference on day 16 [45].

Drought stress in wheat has been analysed by two combined techniques to try and improve detection rates. Moshou [46] uses least squares support vector machine (LSSVM) to try and detect drought stress. Wheat plants were studied in a glasshouse, and both spectral reflectance and fluorescence were analysed. Fluorescence involves using high intensity light to excite a plant tissue causing it to emit a different wavelength light, which can be used to gain additional biological insight. LSSVM needed to be trained, and 846 data samples were used for this training, whilst 302 data samples were used for the testing stage. For some techniques the size of the dataset and/or number of wavelengths will determine the time taken to analyse the data due to computation time. Therefore, Moshou used six wavelengths—503, 545, 566, 608, 860 and 881 nm. The LSSVM attained 76.3% accuracy for stress leaves and 86.6% accuracy for healthy leaves. However, the study stated that by using a fusion LSSVM model combining spectral and florescence features, the overall accuracy was greater than 99%. Fluorescence is the measure of chlorophyll fluorescence in the leaf to determine physiological changes.

According to Kersting [47] many of these techniques are difficult to use for non-machine learning or data mining experts because the hyperspectral data needs pre-processing or adapting (i.e. finding the leaves or using select wavelengths). In addition, the other techniques apart from [44] do not analyse lots of plants over several days. This is an important factor to consider for plant phenotyping when there is a lot of data to analyse. Kersting claims to have the first Artificial Intelligence technique for drought stress prediction using hyperspectral data. A novel approach is developed which includes a predictive technique for drought that does not adapt the data or reduce the size. Kersting demonstrates the approach in a Barley drought experiment with data collected over a five-week period. The technique used is called Dirichlet aggregation regression (DAR) and it is based on matrix factorisation. First Simplex Volume Maximisation is used to find 50 spectral signatures from the data and classify them. Then, latent Dirichlet aggregation values are estimated before using a Gaussian process over the values to find the drought levels per plant and per time point. Finally, the process predicts the drought-affected plants before there are visible signs. Based on a five-week barley experiment, prediction of drought occurred 1.5 weeks before visible signs appeared. A comparison of runtimes between SiVM and DAR was assessed and resulted in a runtime of 30 min for parallelized SiVM, versus only several minutes using the DAR model. This demonstrates that developing custom analysis techniques can outperform (either in computation time, required assumptions, ease of use, or final accuracy) the direct application of existing approaches.

## Hyperspectral data capture and software

Hyperspectral data is large in size, especially when multiple plants are imaged for several days. A scan of a single plant could easily be around a gigabyte in size. If the whole spectrum range is analysed then the process will take considerably longer than selecting several wavelengths to analyse. However, there is a lot of information contained in the data, which could be valuable. The researcher must make decisions about how much spectral resolution to use, and how much to discard. If your camera collects 800 spectral bands, you must ask yourself if you need all 800 or whether binning into 400 or 200 etc. bands is sufficient. This is analogous to using something like JPEG compression for RGB images. This compression creates smaller file sizes, at the expense of destroying image information permanently (particularly colour information). Storing fewer spectral bands results in smaller file sizes, and reduces the complexity of the data analysis, at the expense of throwing away potentially important colour properties. Polder et al. [48] explore the calibration and characterisation of spectrographs captured using three system set ups. The experiments look at the different types of noise and signal-to-noise ratio. The experiments also determined that to an extent binning can occur without loss of information by calculating the resolution, the spectral range and the amount of pixels.

### Hyperspectral camera set-up

Prior to analysis, the hyperspectral data needs to be calibrated to ensure the images produced are adjusted due to the colour of lighting present; the camera software may have this option, but if it does not then the data can be calibrated after it is captured. The lighting is calibrated using a known white balance target, which is imaged by the camera system. This target will reflect a known percentage of light over the spectrum, for example 99% across the entire working spectrum of the camera. Non-uniformity of illumination can be corrected for by dividing the observed data by the captured white balance data [49]. Additionally, the system must be corrected for electrical noise present from the sensor in the absence of light (called dark current). This is usually carried out by taking an image with the camera in the absence of any light, and using the resultant low-level noise readings to adjust future measures.

An important question is how often to carry out a white balance calibration. In a lab setting, it may be appropriate to capture just one white balance target per experiment, assuming the lighting has reached an equilibrium (i.e. the bulbs have fully warmed up). Outside the lab, however, lighting is subject to much more variation. Cloud cover, shadows and time of day can dramatically affect the colour of the incoming light when outside and so very regular white balance readings must be taken to ensure accurate calibration. Careful choice must also be made about the time of day images are captured on, and whether to capture in overcast conditions versus direct sun (which can cause problems with shadows and specular reflection—bright spots on the plants reflecting the illumination source (i.e. the sun) directly). Evenness of illumination should also be considered—does the sensor record a uniform level of brightness across its spatial range? An effect called vignetting can result in pixels towards the edges of the lens appearing darker than those in the centre.

## Conclusions

There has been a significant increase in scientific literature in recent years focusing on detecting stress in plants using hyperspectral image analysis. Plant disease detection is a major activity in the management of crop plants in both agriculture and horticulture. In particular, detecting early onset of stress and diseases would be beneficial to farmers and growers as it would enable earlier interventions to help mitigate against crop loss and reduced crop quality. Hyperspectral imaging is a non-invasive process where the plants are scanned to collect high-resolution data. The technology is becoming more popular since the falling costs of camera production have enabled researchers and developers greater access to this technology. There are various techniques available to analyse the data to detect biotic and abiotic stress in plants, examples of which have been discussed in this review, with a focus on the classification of healthy and diseased plants, the severity of disease and early detection of stress symptoms.

Vegetation and disease indices are increasing in quantity every year. Significant wavelengths combined together can indicate the health or disease status occurring within a specific species. Indices are valuable for detecting specific criteria for vegetation however; the indices are selected with the datasets, species and conditions favourable to the experiments at that time. Some are more general in nature; NDVI, PRI and several other Vegetation Indices will work to find the general health of the plant. But in general, it is harder to take an index designed for plant X and apply it to a dataset for plant Y. This is the motivation behind considering a larger range of wavelengths over the spectrum, which has the potential to yield better results.

## Abbreviations and glossary

ANN: Artificial neural network—neural networks with input vector and output vectors (neurons/nodes) with one or multiple hidden layers of nodes, where all of the layers are fully connected with weights and an activation function
DT: Decision tree has a tree structure form and it is has decision nodes and leaf nodes where the decision nodes have two or more branches and the leaf nodes represent the classification. This is supervised and needs training. The decision rules can become complex as the tree depth increases
Erosion and dilation: Erosion shrinks the foreground object by turning boundary pixels into background pixels if there are more background pixels connected (neighbouring pixels) than foreground pixels. Dilation is the opposite and enlarges the boundary pixels of the foreground object
FLDA: Fishers linear discriminant analysis—project the feature space (n dataset) onto a subspace, dimensionality reduction
MLP: Multilayer perceptron’s are feed forward artificial neural networks (ANN).The MLP is supervised which means it needs a training data set that is labelled [1]
PLSR: Partial least squares regression—using linear regression to find the small set of variables from a large set of predictors by finding the latent variables (covariance of the predictors and variables)
QDA: Quadratic discriminant analysis classifies using a covariance matrix where each class has a unique matrix and therefore has different class density probabilities
SAM: Spectral angle mapper matches the pixel spectra to reference spectra to classify the pixels by calculating the angle between the spectra which are n-dimensional vectors in space [2]
SID: Spectral information divergence compares the divergence between the observed spectra and the reference spectra where the smaller the divergence value the similar the spectra are and if they are larger than a threshold then they are not classified as the reference spectra [3]
SiVM: Simplex volume maximisation selects spectral signatures that are the furthest away from each other to maximise the volume (for example healthy and diseased signatures). Once the signatures have been selected the remaining signatures are assigned to the class they are similar to
SVM: Support vector machine—machine learning process that takes data and splits it into groups/classes based on the training labelled data
LSSVM: Least squares support vector machine
LRDSI: Leaf rust disease severity index
RNIR: Reflectance at NIR (near infrared)
RRED: Reflectance at red
Supervised: Requires the known outcomes for a training dataset, with the training data including inputs and the corresponding expected outputs
Unsupervised: Only the input data is supplied and the training involves the technique learning the underlying structure of the data, there is no correct output
Machine learning: Training the technique to learn rather than using explicit instructions and repeating the process until the objective is reached
RGB: A colour image in the red, green and blue colour space
Multispectral: Several wavelengths that are typically from the visible and/or near infra-red range
Hyperspectral: Hundreds of contiguous narrow bands over a spectral range
Colour binning: Combining wavelengths to reduce the number of wavebands and size of the images
NMF: Non negative matrix factorisation
